# Duration of adjuvant chemotherapy for breast cancer: a joint analysis of two randomised trials investigating three versus six courses of CMF

**DOI:** 10.1038/sj.bjc.6600334

**Published:** 2002-06-05

**Authors:** M Colleoni, H J Litman, M Castiglione-Gertsch, W Sauerbrei, R D Gelber, M Bonetti, A S Coates, M Schumacher, G Bastert, C-M Rudenstam, C Schmoor, J Lindtner, J Collins, B Thürlimann, S B Holmberg, D Crivellari, C Beyerle, R L A Neumann, A Goldhirsch

**Affiliations:** European Institute of Oncology, Milan, Italy; IBCSG Statistical Center, Dana-Farber Cancer Institute and Frontier Science and Technology Research Foundation, Boston, Massachusetts, USA; IBCSG Coordination Center, Bern, Switzerland; Institute of Medical Biometry and Medical Informatics, University Hospital of Freiburg, Freiburg, Germany; The Cancer Council Australia and University of Sydney, Sydney, Australia; Department of Gynaecology, Marien Hospital Essen-Altenessen, Essen, Germany; Department of Gynaecology, University Hospital of Heidelberg, Heidelberg, Germany; Western Sweden Breast Cancer Study Group, Sahlgrenska University Hospital, Göteborg, Sweden; Institute of Oncology, Ljubljana, Slovenia; Anti-Cancer Council of Victoria, Melbourne, Australia; Kantonsspital, St. Gallen, Switzerland; Centro di Riferimento Oncologico, Aviano, Italy; Department of Gynaecology, Kreiskrankenhaus Kronach, Kronach, Germany; Oncology Institute of Southern Switzerland, Lugano, Switzerland

**Keywords:** breast cancer, chemotherapy duration, cyclophosphamide, methotrexate and fluorouracil (CMF), predictive factors

## Abstract

Cyclophosphamide, methotrexate and fluorouracil adjuvant combination chemotherapy for breast cancer is currently used for the duration of six monthly courses. We performed a joint analysis of two studies on the duration of adjuvant cyclophosphamide, methotrexate and fluorouracil in patients with node-positive breast cancer to investigate whether three courses of cyclophosphamide, methotrexate and fluorouracil might suffice. The International Breast Cancer Study Group Trial VI randomly assigned 735 pre- and perimenopausal patients to receive ‘classical’ cyclophosphamide, methotrexate and fluorouracil for three consecutive cycles, or the same chemotherapy for six consecutive cycles. The German Breast Cancer Study Group randomised 289 patients to receive either three or six cycles of i.v. cyclophosphamide, methotrexate and fluorouracil day 1, 8. Treatment effects were estimated using Cox regression analysis stratified by clinical trial without further adjustment for covariates. The 5-year disease-free survival per cents (±s.e.) were 54±2% for three cycles and 55±2% for six cycles (*n*=1024; risk ratio (risk ratio: CMF×3/CMF×6), 1.00; 95% confidence interval, 0.85 to 1.18; *P*=0.99). Use of three rather than six cycles was demonstrated to be adequate in both studies for patients at least 40-years-old with oestrogen-receptor-positive tumours (*n*=594; risk ratio, 0.86; 95% confidence interval, 0.68 to 1.08; *P*=0.19). In fact, results slightly favoured three cycles over six for this subgroup, and the 95% confidence interval excluded an adverse effect of more than 2% with respect to absolute 5-year survival. In contrast, three cycles appeared to be possibly inferior to six cycles for women less than 40-years-old (*n*=190; risk ratio, 1.25; 95% confidence interval, 0.87 to 1.80; *P*=0.22) and for women with oestrogen-receptor-negative tumours (*n*=302; risk ratio, 1.15; 95% confidence interval, 0.85 to 1.57; *P*=0.37). Thus, three initial cycles of adjuvant cyclophosphamide, methotrexate and fluorouracil chemotherapy were as effective as six cycles for older patients (40-years-old) with oestrogen-receptor-positive tumours, while six cycles of adjuvant cyclophosphamide, methotrexate and fluorouracil might still be required for other cohorts. Because endocrine therapy with tamoxifen and GnRH analogues is now available for younger women with oestrogen-receptor-positive tumours, the need for six cycles of cyclophosphamide, methotrexate and fluorouracil is unclear and requires further investigation.

*British Journal of Cancer* (2002) **86**, 1705–1714. doi:10.1038/sj.bjc.6600334 www.bjcancer.com

© 2002 Cancer Research UK

## 

The question of duration of adjuvant chemotherapy for breast cancer has been directly addressed in several trials. Most of these were small and, therefore, unsuitable for detecting differences of modest magnitude (Bonadonna *et al*, 1987; Levine *et al*, 1990; Senn and Jungi, 1984; Falkson *et al*, 1989; Henderson *et al*, 1986). A meta-analysis of six such trials showed that a shorter treatment duration (6 months) was as effective as a longer duration therapy (12–24 months) (Early Breast Cancer Trialists' Collaborative Group, 1998a). The International Breast Cancer Study Group (IBCSG) Trial V investigated the role of a single cycle of cyclophosphamide, methotrexate, and 5-fluorouracil (CMF) immediately after the operation as compared with six or seven cycles of the same treatment for patients with node-positive disease (Ludwig Breast Cancer Study Group, 1988). Although a single cycle of perioperative chemotherapy improved outcome compared with no adjuvant chemotherapy in node negative disease (Ludwig Breast Cancer Study Group, 1989), such treatment was found to be less effective than a longer duration CMF in node positive disease (Ludwig Breast Cancer Study Group, 1988). Without considering subgroups, these results suggest that the optimal duration of adjuvant CMF therapy for breast cancer is more than one but not more than six 28-day cycles. The duration of a typical anthracycline-containing regimen tested in several adjuvant trials is 3 months (administered once every 3 weeks for four courses). This regimen yielded similar disease-free and overall survival results compared with six courses of CMF (Fisher *et al*, 1990, 2000).

Recently, the German Breast Cancer Study Group (GBSG) reported the 10-year follow-up results of a randomised trial in 481 node-positive patients comparing three versus six cycles of day 1 and 8 i.v. CMF (modified Bonadonna regimen) with or without tamoxifen (Sauerbrei *et al*, 2000). No difference in overall survival or event-free survival between the two durations of CMF was found. The International Breast Cancer Study Group (IBCSG) Trial VI compared three versus six cycles of initial ‘classical’ CMF (oral cyclophosphamide) with or without three additional single cycles of ‘reintroduction’ CMF for 1554 premenopausal women with node-positive breast cancer. At 5 years' median follow-up, the IBCSG previously reported that three cycles of adjuvant CMF chemotherapy yielded a shorter disease-free survival compared with longer duration treatment, based on the entire trial population (International Breast Cancer Study Group, 1996). However, the increased risk of relapse with CMF×3 was marked for women aged less than 40 years and for patients with ER-negative tumours (International Breast Cancer Study Group, 1996); patients older than 40 years had similar disease free survival times. Furthermore, the patients assigned to three cycles showed more rapid adjustment in self-reported quality of life (Hürny *et al*, 1996) and had significantly less objective and subjective toxicity than those assigned longer duration of therapy (International Breast Cancer Study Group, 1996). To further clarify the role of duration of adjuvant chemotherapy, we report a joint analysis using individual data based on the recently published results of the GBSG trial and updated results of IBCSG Trial VI with a median follow up of 7.9 years. Both studies included two additional arms (two by two designs) that are not the subject of this report.

## PATIENTS AND METHODS

### Description of trials

From July 1986 to April 1993, 1554 pre- and perimenopausal breast cancer patients with node-positive disease were randomised in IBCSG Trial VI in a two by two factorial design to receive the following: (A) cyclophosphamide, methotrexate, and fluorouracil for six consecutive cycles on months 1 to 6 (CMF×6); (B) CMF×6 plus three single cycles of reintroduction CMF given on months 9, 12 and 15; (C) CMF for three consecutive cycles on months 1 to 3 (CMF×3); or (D) CMF×3 plus three single cycles of reintroduction CMF given on months 6, 9 and 12. The randomization was stratified according to participating institution, type of surgery (mastectomy *vs* breast-conserving procedure with breast irradiation), and oestrogen receptor (ER) status (negative *vs* positive). The median follow-up was 7.8 years for disease free survival and 7.9 years for overall survival. The protocol required that the adjuvant chemotherapy begin within 6 weeks of surgery and consist of CMF (cyclophosphamide 100 mg m^−2^ orally days 1-14, methotrexate 40 mg m^−2^ i.v. days 1 and 8, 5-fluorouracil 600 mg m^−2^ i.v. days 1 and 8, repeated every 28 days). Oestrogen receptor concentrations in the primary tumours were determined by standard methods and concentrations ⩾10 fmol mg^−1^ of cytosol protein were considered positive; lower values negative. Surgery of the primary tumour was either a total mastectomy with axillary clearance, or a lesser procedure (quadrantectomy or lumpectomy) with axillary lymph node dissection. For women treated with breast conserving surgery, radiotherapy was mandatory and had to be postponed until the end of the initial phase of chemotherapy (3 or 6 cycles).

From 1984 to 1989, 481 breast cancer patients were randomised in the GBSG Trial in a two by two factorial design to receive either three or six cycles of CMF and to receive two years of hormonal therapy with tamoxifen or no hormonal therapy. The trial design was the following: (A) CMF×3; (B) CMF×3+tamoxifen; (C) CMF×6; and (D) CMF×6+tamoxifen. At the beginning of the study, both pre- and postmenopausal patients were randomised to all four arms of the study. However, starting in December 1986, premenopausal patients were only randomised to treatment arms A and C. Chemotherapy was administered according to the modified Bonadonna CMF regimen, which consisted of 500 mg m^−2^ cyclophosphamide, 40 mg m^−2^ methotrexate, and 600 mg m^−2^ fluorouracil administered intravenously on days 1 and 8 of a 4-week treatment period. Chemotherapy started within 36 h after surgery. Hormonal treatment consisted of a daily dose of 3×10 mg tamoxifen orally over 2 years, starting after the third cycle of CMF. The median follow-up was 9.1 years for disease free survival and 10.0 years for overall survival.

### Statistical methods

A joint analysis of the two studies was performed to compare the effectiveness of CMF×3 and CMF×6. Only data in arms A and C from both studies were used in the analysis (CMF×3 only and CMF×6 only treatment arms: Seven hundred and thirty-five patients from the IBCSG trial and 289 patients from the GBSG trial). Patient characteristics according to randomised treatment are presented for each of the trials in Table 1

**Table 1:**
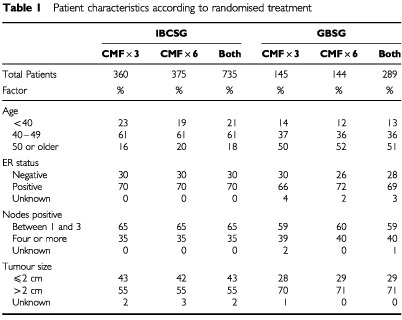
Patient characteristics according to randomised treatment

. Disease-free survival (DFS) was defined as the time from surgery to relapse, second malignancy, or death without relapse, whichever occurred first. Overall survival (OS) was defined as the time from surgery to death from any cause. Five-year DFS rates were calculated for each treatment group by trial, as well as by age group (<40, ⩾40), by ER status (negative, positive), and, for the IBCSG trial only, by both age group and ER status. Risk ratios (RR) with pointwise confidence intervals for DFS comparing CMF×3 to CMF×6 were calculated based on Cox models stratified by clinical trial without inclusion of covariates (Cox, 1972). An RR greater than one demonstrated that the risk of disease relapse for patients treated with CMF×3 was higher than for patients treated with CMF×6. Chi-square Wald statistics for the null hypothesis that the RR equals one were calculated. Two-sided *P*-values less than 0.05 from this test were considered significant. Nine cases in the GBSG trial did not have ER values, so they are not included in the DFS risk ratios by ER status.

**Figure 1:**
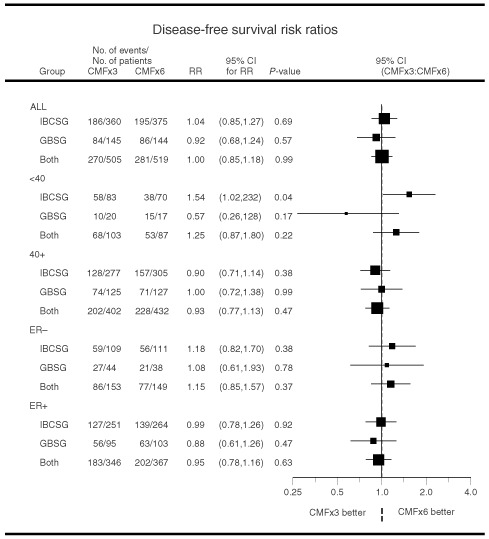
Risk ratios comparing three cycles *vs* six cycles of CMF overall and according to age (<40, ⩾40) and ER status (negative, positive).

A graph of 95% confidence intervals (CI) for the RR overall and by age and ER status is presented in Figure 1

. The size of the boxes on the figure represents the amount of information available from each subset (the larger the box, the more information provided). Specifically, the box size is inversely proportional to the standard error of the estimate of the natural log of the RR. Kaplan–Meier curves of DFS for the two trials by treatment arm and also by age group were generated (Kaplan and Meier, 1958). Kaplan–Meier curves of OS by cooperative group and treatment arm were created.

To describe the relationship between age and the magnitude of treatment difference we used subpopulation treatment effect pattern plots (STEPPs). This statistical method provides estimates of the hazards ratios computed by the Cox model fitted on overlapping subpopulations of patients, where the subpopulations are defined so that they contain patients having increasing age (Bonetti and Gelber, 2000). A second implementation of STEPP describes the 5-year DFS estimated for each treatment group within each of the overlapping subpopulations (Bonetti and Gelber, 2001).

### Differences between the two CMF 3 *vs* 6 trials

The administration of CMF regimens differed between the trials. Patients in the GBSG trial received i.v. CMF (modified Bonadonna regimen), while oral cyclophosphamide and i.v. methotrexate and fluorouracil were given to patients in the IBCSG trial.

Patients in the GBSG study were randomised in most cases following three cycles of CMF to avoid including early drop-outs in the data analysis. Patients in the IBCSG study were randomised prior to the first cycle of CMF. To maintain consistency between the two trials, surgery date was used as a common starting date for calculation of time to event endpoints (age, DFS, and OS). In the IBCSG study, follow-up information for DFS and for OS were obtained from study forms submitted to the data management center. In the GBSG study, DFS and OS were based on clinic data; however, for some patients or clinics, contacts were lost after some time. In these cases, information about survival status was requested from the corresponding registration office. This additional information was used for OS only.

In the published report on the GBSG study, patients with ER values of 20 fmol mg^−1^ or greater were considered ER-positive. ER values of 10 fmol mg^−1^ or greater were ER-positive in the IBCSG study. The joint analysis considered ER values of 10 fmol mg^−1^ or greater to be ER-positive.

Timing of scheduled follow-up visits differed between the two trials. In the IBCSG trial, the patients were seen every 3 months for the first 2 years, every 6 months for the next 3 years, and then annually thereafter. Patients were scheduled to be seen more frequently in the GBSG trial. They were seen every 3 months for the first 2 years after the operation, every 4 months for the following 3 years, every 6 months for the following 2 years, and then annually thereafter. Median follow-up was 7.8 years for disease free survival in the IBCSG trial and 9.1 years in the GBSG trial. Of the 735 IBCSG patients, 381 had an event and of the 289 GBSG patients, 170 had an event. Median overall follow-up for the IBCSG trial was 7.9 years and 10.0 years for the GBSG trial. 254 of the 735 IBCSG patients died, and 143 of the 289 GBSG patients died thus far.

As shown in Table 1, 51% of patients in the GBSG trial were at least 50 years of age, while only 18% of patients in the IBCSG trial were 50 or older. The majority of patients in both the GBSG and IBCSG trials had ER-positive tumours (69 and 70%, respectively). Most patients in both trials had between one and three positive nodes (59 and 65%, respectively). A higher percentage of patients in the GBSG trial had tumours larger than two centimeters, 71% compared with 55% in the IBCSG trial. All of the GBSG patients and 71% of the IBCSG patients had mastectomies.

## RESULTS

**Figure 2:**
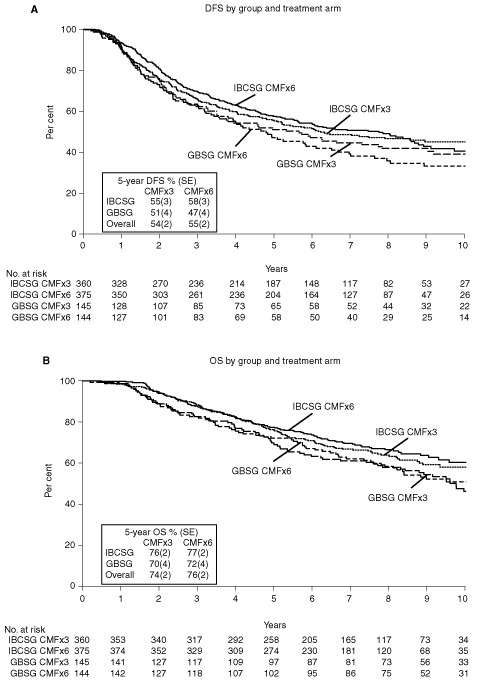
Kaplan–Meier plots of disease-free survival according to cooperative group and treatment (**A**) and Kaplan–Meier plots of overall survival according to cooperative group and treatment (**B**).

Across all patients from both trials, DFS RRs of CMF×3 v CMF×6 revealed no difference between treatment groups (Figure 1, top). The RR for the 1024 patients was 1.00, with a DFS Cox model *P*-value of 0.99 indicating no DFS difference between the two treatments. Graphically, Figure 2A

shows the similar Kaplan–Meier DFS curves for the two treatments within each of the individual trials. The 5-year DFS per cents±s.e. across both trials were 54±2% for three cycles and 55%±2% for six cycles (absolute difference [95% CI], −1% [−7% to +6%]). The RR of OS for the 1024 patients was 1.10 with 95% CI 0.90 to 1.34 (*P*=0.35). Figure 2B shows Kaplan–Meier curves for OS. The five-year OS per cents±s.e. across both trials were 74±2% for three cycles and 76±2% for six cycles (absolute difference [95% CI], −1% [−7% to +4%]).

**Figure 3:**
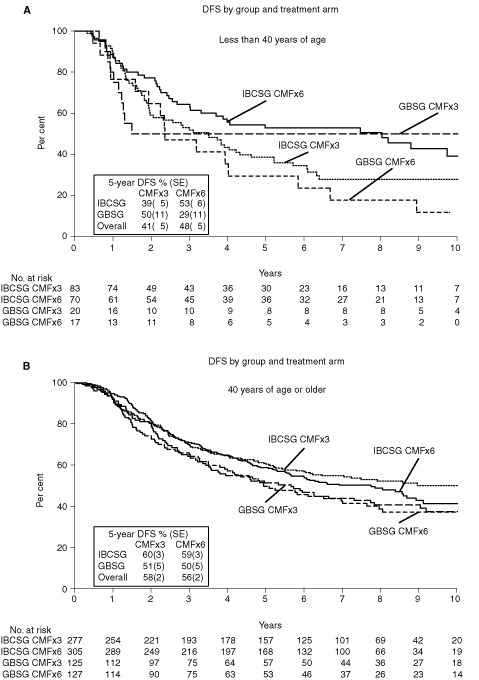
Kaplan–Meier plots of disease-free survival according to cooperative group and treatment for patients less than 40 years of age (**A**) and for patients 40 years of age or older (**B**).

**Figure 4:**
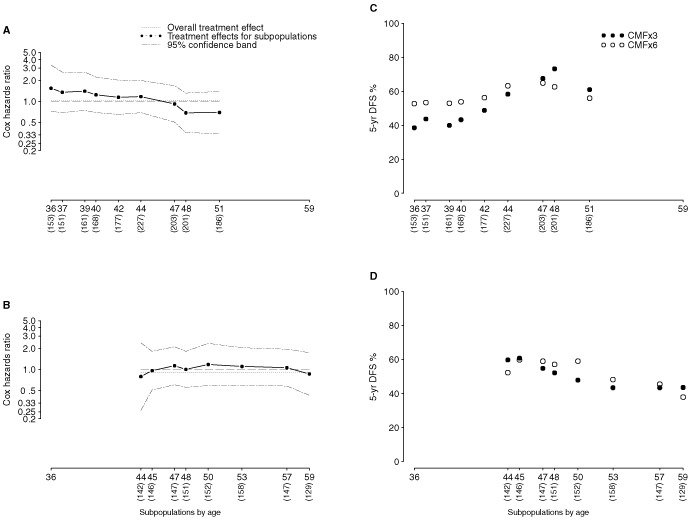
Subpopulation Treatment Effect Pattern Plots (STEPPs) according to age for the IBCSG trial (**A** and **C**) and for the GBSG trial (**B** and **D**).

For patients who were less than 40 years old (regardless of ER status), results suggested an increased risk of relapse for patients on CMF×3 versus CMF×6. Across both trials, for patients younger than 40 years of age the RR was 1.25 with 95% CI 0.87 to 1.80 (*P*=0.22; Figure 1). For these patients, the 5-year DFS was 41±5% for CMF×3 compared with 48±5% for CMF×6 (Figure 3A

). Five-year OS was 66±5% for CMF×3 compared with 70±5% for CMF×6 for patients younger than 40 years of age. The STEPP analyses for the IBCSG trial (Figures 4A,C) graphically showed that differences in treatment effect are likely to occur across the continuum of age, with CMF×3 compromising DFS for patients <40 compared with CMF×6. Both the Cox model implementation of STEPP (Figure 4A

) and the plot showing 5-year DFS per cents (Figure 4C) illustrate improved outcome for younger patients in the CMF×6 group. Because there were so few patients under 40 in the GBSG trial, this effect could not be investigated in Figures 4B and 4D.

The results for all patients with ER-negative tumours (*n*=302) also showed a possible increased risk for the CMF×3 group (Figure 1, RR=1.15; 95% CI=0.85 to 1.57; *P*-value=0.37). Five-year DFS Kaplan–Meier estimates for this patient subpopulation were 48±4% for three cycles compared with 54±4% for six cycles of CMF. Five-year OS Kaplan–Meier estimates were 60±4% for three cycles compared with 71±4% for six cycles of CMF. Both the IBCSG and GBSG studies showed that CMF×3 may not be sufficient for ER-negative patients (RR 1.18 and 1.08, respectively, Figure 1), although the results were not statistically significant.

**Figure 5:**
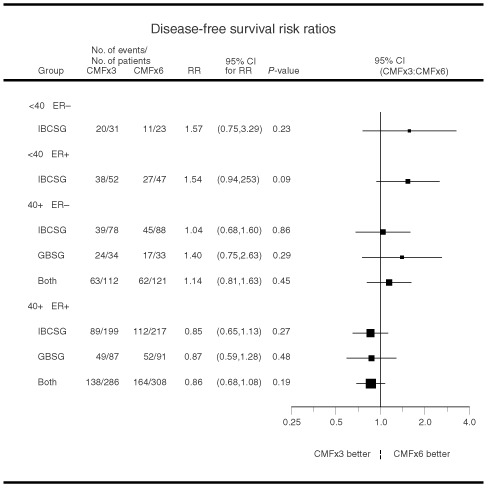
Risk ratios comparing three cycles *vs* six cycles of CMF according to subpopulations defined by both age (<40, ⩾40) and ER status (negative, positive). Because there were so few GBSG patients who were younger than 40, only IBCSG data were used for these calculations.

The increased risk of relapse in the CMF×3 group for patients under 40-years-old was observed both for patients with ER-positive and for those with ER-negative tumours. The RR for patients less than 40-years-old with ER-negative tumours was 1.57 and for patients less than 40 with ER-positive tumours was 1.54. Neither RR was statistically significantly different from one. Because there were so few GBSG patients who were younger than 40, only IBCSG data were used for these calculations (Figure 5

).

For patients at least 40-years-old with ER-positive tumours, use of three cycles did not increase the risk of relapse compared with six cycles of CMF (Figure 5, *n*=594, RR=0.86, 95% CI=0.68 to 1.08, *P*-value=0.19). This result was consistent for each of the trials. The IBCSG trial had an RR of 0.85 with 95% CI 0.65 to 1.13 and the GBSG trial had an RR of 0.87 with 95% CI 0.59 to 1.28. Across both trials, the 5-year DFS±SE was 61±3% for three cycles of CMF and 57±3% for six cycles of CMF (absolute difference [95% CI], +4% [−4 to +12%]). Five-year OS±SE was 83±2% for three cycles of CMF and 79±2% for six cycles of CMF for patients at least 40 years old with ER-positive tumours (absolute difference [95% CI], +4% [−2 to +11%]).

In general, formal tests for interaction between the duration of CMF and either age or ER status alone were not statistically significant (Figure 1; *P*=0.17 for age and *P*=0.33 for ER). Among patients with ER-positive tumours, however, the longer duration treatment provided benefit for younger women but not for older women (Figure 5; *P*-value for interaction=0.03).

## DISCUSSION

There is high quality evidence that adjuvant cytotoxic chemotherapy delays relapse and prolongs survival for patients with early breast cancer (Early Breast Cancer Trialists' Collaborative Group, 1998a). As in other areas of medicine, once primary efficacy has been demonstrated research efforts move to evaluate secondary questions addressing optimization of the balance between the benefits of such treatment and its subjective and objective costs. One such question concerns the optimal duration of adjuvant chemotherapy. In premenopausal women, cytotoxic therapy is thought to exert its effects both by direct tumour cell kill and by an endocrine mechanism secondary to suppression of ovarian function (Pagani *et al*, 1998). The extent to which chemotherapy may exert such an endocrine effect will depend on the type of chemotherapy, the age of the patient (known to influence the probability of chemotherapy-induced amenorrhea; Pagani *et al*, 1998; Goldhirsch *et al*, 1990), and the hormone receptor expression of the tumour (endocrine therapy will be more important in receptor-positive disease; Scottish Cancer Trials Breast Group and ICRF Breast Unit, 1993). Effective alternative forms of endocrine therapy such as tamoxifen (Early Breast Cancer Trialists' Collaborative Group, 1998b), ovarian ablation, and medical suppression of ovarian function are available, so the optimal duration of cytotoxic therapy may also depend on whether or not such treatments are used. Endocrine non-responsive disease is controlled only by the direct cytotoxic effects of chemotherapy and the benefit of longer duration chemotherapy might best be studied in this setting in the absence of endocrine therapies.

Several trials examine regimens which differ in duration of therapy but also in the drugs given. In these trials the effects of duration and choice of drug are inextricably confounded. They reach varying conclusions about treatment duration. Thus, one of the most frequently used anthracycline-containing adjuvant therapy programs, four cycles of intravenous doxorubicin (Adriamycin®) and cyclophosphamide (AC) combination given once every 3 weeks, is administered entirely within 63 days. In a direct comparison, six courses of classical CMF (154 days) and four courses of AC yielded similar results despite the different durations (Fisher *et al*, 1990, 2000). Likewise a short but complex 16-week regimen (including a continuous administration of cytotoxics during the entire period of treatment) yielded results marginally superior to those seen with six courses of cyclophosphamide, doxorubicin and 5-fluorouracil (CAF) (Fetting *et al*, 1998). On the other hand, the US Intergroup trial of the addition of 4 cycles of paclitaxel (Taxol®) following four cycles of AC demonstrated a small but significant improvement in disease-free and overall survival using the longer, different regimen (Henderson *et al*, 1998). This improvement was seen almost exclusively among patients with ER-negative tumours who did not receive tamoxifen.

Evidence already available defines broad limits within which the optimal duration of adjuvant CMF therapy may be expected to be found. The EBCTCG overview found no advantage in extending therapy beyond about 6 months (Early Breast Cancer Trialists' Collaborative Group, 1998a). International Breast Cancer Study Group Trial V demonstrated that a single peri-operative cycle of CMF was less effective than a course of six or seven cycles of CMF (Ludwig Breast Cancer Study Group, 1988) though a single cycle did afford some therapeutic benefit (Ludwig Breast Cancer Study Group, 1989). There is little evidence directly comparing treatment regimens of between more than one and 6 months duration.

There are additional reasons to investigate the question of treatment duration. Subjectively, a course of three cycles of CMF was better tolerated, and associated with more rapid improvement in quality of life than 6 cycles (Hürny *et al*, 1996). Less toxicity was observed with three cycles of CMF in the GBSG study (Schumacher *et al*, 1994). Three cycles of CMF chemotherapy was also shown to be effective in addition to tamoxifen therapy in postmenopausal patients, with minimal adverse effect on quality of life (Castiglione-Gertsch *et al*, 2000). Finally, shorter duration treatments are less costly than longer durations of the same agents.

In this joint analysis we investigated the issue of duration of CMF chemotherapy by choosing two trials, conducted by the IBCSG (International Breast Cancer Study Group, 1996) and the GBSG (Sauerbrei *et al*, 2000) that used the same three agents in similar schedules and common study questions, though with different routes of cyclophosphamide administration. Both studies also addressed other questions. In this analysis we avoided these potential confounding treatment factors, which included the use of tamoxifen (GBSG) and additional cycles of chemotherapy (IBCSG), by selecting only the data from patients randomised to receive three or six cycles of CMF in each study.

The two studies jointly reviewed here demonstrate that for the overall population and especially for older patients (40 years and above) with hormone-receptor positive tumours three cycles of CMF chemotherapy is nearly identical to six cycles, even in the absence of specific additional endocrine therapy. This is consistent with the EBCTCG overview (Early Breast Cancer Trialists' Collaborative Group, 1998a), which found a real but relatively small benefit for any chemotherapy in older patients with ER-positive tumours. Such patients are now routinely treated with tamoxifen or ovarian ablation in addition to chemotherapy, presumably further reducing the need for more prolonged cytotoxic therapy. The evidence to support the shorter duration of chemotherapy compared with the longer standard duration is, however, unreliable for younger patients and for those with ER-negative disease, and the longer duration treatment might be beneficial for these cohorts.

Since the current analysis was performed, Maass (2000) have presented preliminary data from a further German trial (GABG III) comparing three versus six cycles of adjuvant CMF and confirming no overall difference between the treatment groups. Based on 789 patients with one to nine axillary lymph nodes involved at a median follow-up of 35 months, no difference in disease-free (*P*=0.34) or overall survival (*P*=0.17) was found (three versus six cycles: percent relapsing, 31.3 *vs* 30.4%; per cent dead, 14.8 *vs* 12.8%). Data for subgroups according to age and steroid hormone receptor status of the primary tumour were not presented, but no significant treatment difference was found for any subgroup. As far as we know, the GBSG, IBCSG and GABG trials are the only randomised studies evaluating three versus six cycles of CMF, and all demonstrated no overall difference between treatment arms.

We previously published data from IBCSG Trial VI according to amenorrhea suggesting that the endocrine effects of chemotherapy alone are insufficient for the younger age group with ER/PgR positive tumours (Pagani *et al*, 1998). In this study women who experienced amenorrhea had a significantly better DFS than those who did not. DFS differences between amenorrhea categories were larger for patients with ER/PgR positive tumours. The role of chemotherapy induced amenorrhea appeared evident also when outcome was analyzed in a population of 314 very young (<35 years) premenopausal patients enrolled in trials of adjuvant chemotherapy (Aebi *et al*, 2000). The worst prognosis was observed for younger patients with ER-positive tumours who did not achieve amenorrhea. As indicated by the STEPP plots for the current joint analysis, differences in treatment effect are likely to occur across the continuum of age. Thus, clinical decisions concerning the use of three rather than six courses of CMF might be based on assessment of likelihood for chemotherapy-induced amenorhea rather than on a fixed age cut-point.

The results of subgroup analyses should be treated with caution, especially because some of the subgroups had small sample sizes. However, there is some biological rationale for expecting benefit of longer duration therapy in the subpopulation of patients with ER-negative tumours. Such tumours have a more rapid cell proliferation and are associated with a higher risk of relapse despite adjuvant chemotherapy. Prolonged duration of chemotherapy may, therefore, be particularly relevant to inhibit the growth of tumours that are not susceptible to the effects of endocrine therapies due to lack of ER.

As the risk of relapse increases (higher number of positive nodes) the likelihood that an ER-positive phenotype has a proportion of chemotherapy responsive cells also increases. Therefore, although the overall cohort of older patients with ER-positive tumours was safely treated with the shorter duration of chemotherapy, it is possible that the longer duration of chemotherapy provides a modest benefit for patients with ER-positive tumours at high risk of relapse especially in the absence of tamoxifen (Henderson *et al*, 1998; Goldhirsch *et al*, 2000).

Historically, patients were classified as having ER-negative (<10 fmol mg^−1^ cytosol protein or <10% of positive cells) and ER-positive (⩾10 fmol mg^−1^ cytosol protein or ⩾10% of positive cells) tumours to facilitate prediction of response to endocrine therapies. However, there is recent evidence that tumours with less than 10% of weakly positive cells still may experience tumour response, compared with those who had no detectable ER staining (Harvey *et al*, 1999). These data lead to the conclusion that duration of chemotherapy might be tested best in patients with ER-absent tumours, where the cytotoxic rather than endocrine effects of chemotherapy might be even larger. Although this is a small subset compared with the entire breast cancer population, it might represent a group particularly relevant for tailored treament investigations of adjuvant chemotherapy questions, especially in the absence of confounding endocrine therapies.

We conclude that patients with potentially endocrine responsive node-positive disease who are over 40 years of age can be adequately treated with three rather than six cycles of CMF especially if it is followed by tamoxifen. The reduction in the amount of chemotherapy would significantly reduce subjective and objective toxicity. It is difficult to draw firm conclusions for younger patients based on this analysis because there are so few younger patients, particularly in the GBSG trial, and chemotherapy was the only adjuvant treatment. Perhaps three cycles of CMF would be sufficient also for women aged under 40 with hormone receptor positive, potentially endocrine responsive node-positive disease if CMF were followed by effective endocrine therapy. Indeed recent studies (Jakesz *et al*, 1999; Roche *et al*, 2000; Boccardo *et al*, 2000) show endocrine therapy alone with the combination of GnRH analogue plus tamoxifen is equally effective or superior to chemotherapy alone, clearly implying that the endocrine component of any chemoendocrine therapy provides the dominant effect for these patients.

For women of any age with tumours that do not express any steroid hormone receptors (ER-absent) (a relatively small subgroup of patients), the issue of adjuvant chemotherapy duration requires further study, but our results do not suggest that adjuvant CMF can safely be reduced to three cycles in these women.
